# Talus osteoid osteoma misdiagnosed as ankle synovitis: A case report in rehabilitation therapy

**DOI:** 10.1097/MD.0000000000040682

**Published:** 2024-11-29

**Authors:** Jincheng Xu, Yan Zhang, Shuming Yang

**Affiliations:** a Department of Rehabilitation Medicine, Affiliated Huishan Hospital of Xinglin College, Nantong University, Wuxi Huishan District People’s Hospital, Wuxi City, Jiangsu Province, China; b Department of Rehabilitation Therapy, Wuxi Central Rehabilitation Hospital, Wuxi City, Jiangsu Province, China.

**Keywords:** ankle synovitis, multidisciplinary teamwork, osteoid osteoma, physical therapy, talus

## Abstract

**Rationale:**

Osteoid osteoma, accounting for approximately 10% of benign bone tumors, is predominantly found in long bones and rarely in the foot bones, such as the talus. Its nonspecific symptoms, such as nocturnal pain and swelling, often lead to misdiagnosis, especially when it mimics conditions like ankle synovitis.

**Patient concerns:**

A 27-year-old male presented with persistent pain and swelling in his left ankle following an injury at the gym. Initial treatments, including arthroscopic debridement, failed to resolve his symptoms.

**Diagnoses:**

Comprehensive evaluation using magnetic resonance imaging and computed tomography scans, coupled with input from a multidisciplinary team of specialists, confirmed the diagnosis of ankle osteoid osteoma.

**Interventions:**

Following the confirmation of the diagnosis, the patient underwent surgical interventions, including peripheral nerve transfer, joint debridement, resection of calcaneal and talus lesions, and tendon release. These procedures were designed to address the underlying tumor and restore joint function.

**Outcomes:**

After surgical intervention, the patient experienced substantial relief from pain and significant improvement in functional recovery. Postoperative rehabilitation further facilitated the restoration of mobility and strength, with no recurrence of symptoms observed during follow-up.

**Lessons:**

This case highlights the diagnostic complexity of ankle osteoid osteoma and underscores the importance of a multidisciplinary approach. Rehabilitation therapists play a crucial role in managing such conditions, ensuring optimal patient outcomes through functional assessment and progress monitoring. Timely and accurate diagnosis is essential for effective treatment and improved patient quality of life.

## 1. Introduction

The confusion in diagnosing ankle osteoid osteoma (OO) and ankle synovitis arises from their overlapping clinical presentations and imaging findings.^[1]^ OO is a common benign bone tumor that can show signs like inflammatory synovitis, like joint effusion, soft-tissue swelling, ankle pain, and limited joint motion.^[2]^ This resemblance can lead to diagnostic challenges, especially when the tumor is located intracapsularly.^[3,4]^ A study by Bauer et al (1991)^[5]^ highlighted this issue, revealing a significant delay in diagnosis, with an average of 24 months from initial presentation to accurate identification. Radiographs in these patients were frequently inconclusive, showing either negative results or only secondary changes. Similarly, OO is a condition characterized by cartilaginous nodules within the synovium, is rare in smaller joints such as the ankle.^[6]^ As noted by Sedeek et al (2015),^[7]^ its diagnosis usually requires a comprehensive approach involving a detailed history, clinical and physical examination, and radiographic evaluation. This article describes the diagnostic trajectory of a patient with ankle OO from initial suspicion to definitive diagnosis, highlighting the critical role of multidisciplinary collaboration. This case report follows the CARE Guidelines.^[8]^

## 2. Case presentation

**Figure 1.**
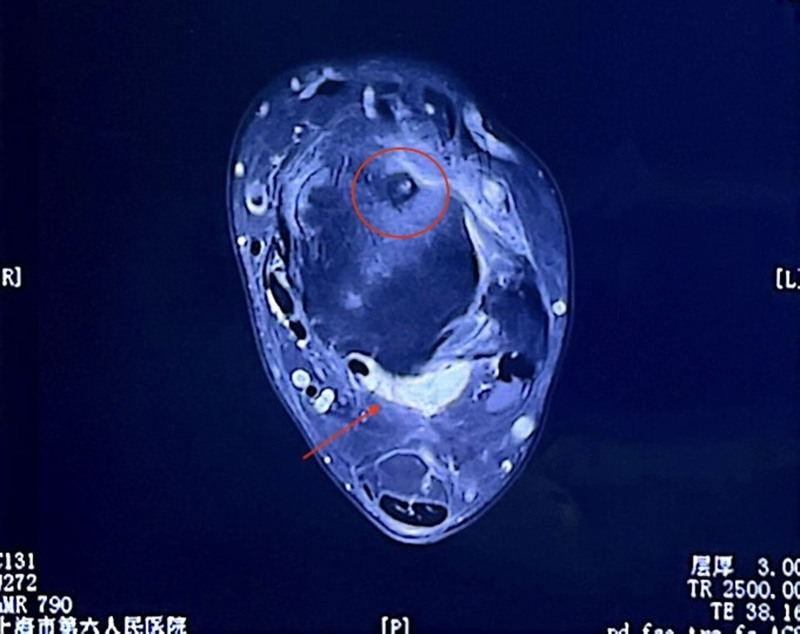
MRI demonstrated dorsal swelling adjacent to the neck of the left talus, accompanied by mild synovitis, posterior subtalar joint effusion, and cystic alterations in the lipoma of the left calcaneal region. MRI = magnetic reservoir imaging.

The case involves a 27-year-old male who initially experienced pain in his left ankle following a gym incident 4 years ago. The primary symptoms included swelling, chronic pain, and restricted motion in the ankle, which progressively worsened over time, particularly at night. The pain was not specifically linked to physical activity, and the patient frequently relied on medications such as etoricoxib and celecoxib for relief. Two years after the initial incident, due to persistent pain and swelling, a magnetic resonance imaging (MRI) scan was conducted. The results showed edema, dorsal swelling around the neck of the left talus, mild synovitis, subtalar effusion in the back, and possible cystic changes in the left calcaneal lipoma (Fig. 1). Initially diagnosed with synovitis in the left ankle, the patient underwent occlusion therapy, but the pain returned within 3 days.

**Figure 2.**
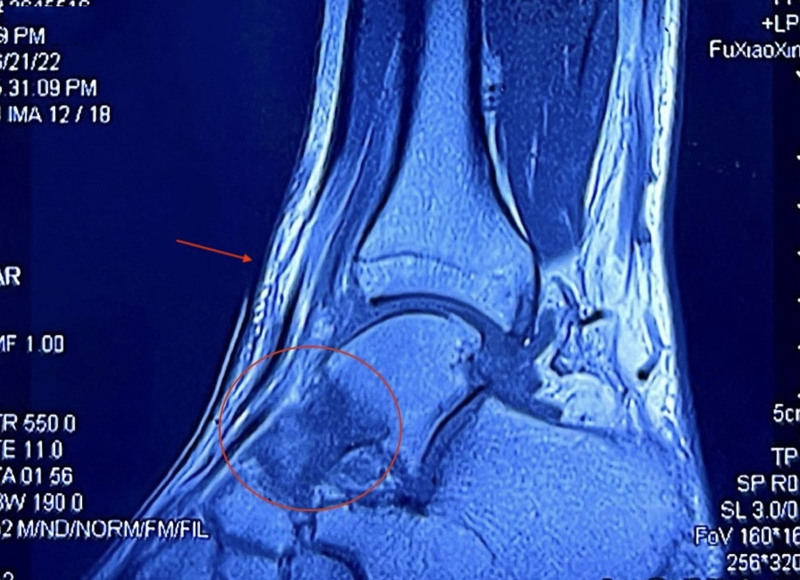
MRI demonstrated talar bone marrow edema, with anterior subcutaneous soft-tissue swelling, and minor joint effusion. MRI = magnetic resonance imaging.

**Figure 3.**
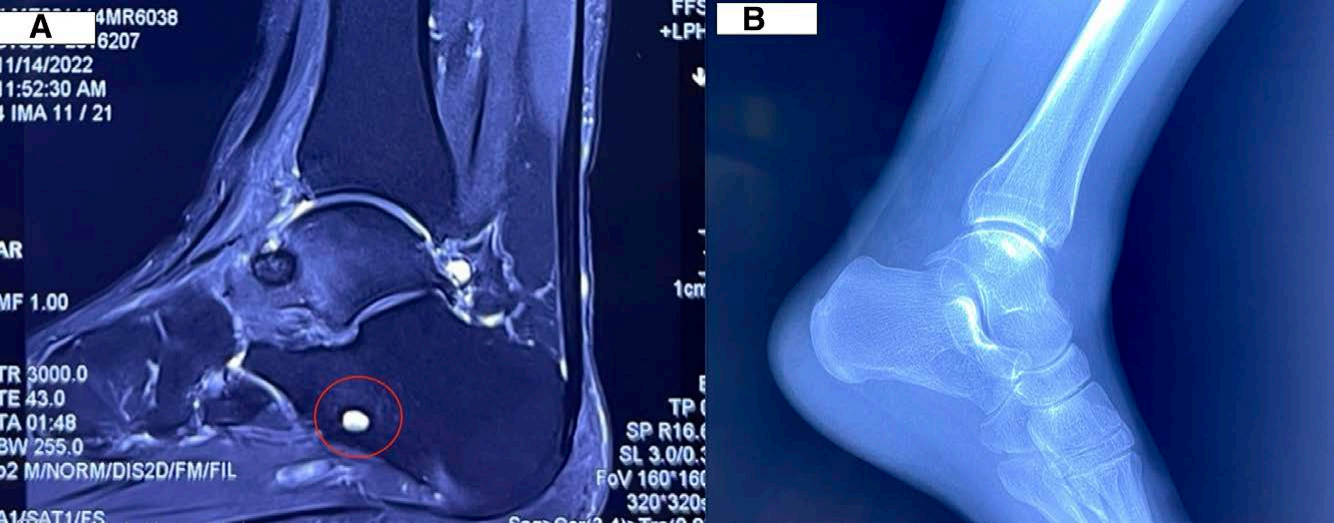
(A) MRI demonstrated a postoperative calcaneal sinus cyst of the left ankle. (B) X-ray imaging revealed small bone hyperplasia at the lower end of the left tibia and the posterior border of the left calcaneal tuberosity. MRI = magnetic resonance imaging.

Subsequently, the patient underwent arthroscopic debridement of the ankle joint at another hospital. Although there was temporary symptom relief post-surgery, the pain intensified again within a month, especially at night. A follow-up MRI 2 weeks after the operation revealed bone marrow edema in the talus, anterior subcutaneous soft-tissue swelling, and minor joint effusion (Fig. 2). Six months later, an MRI revealed a postoperative calcaneal sinus cyst in the left ankle (Fig. 3A). X-ray imaging revealed small bone hyperplasia at the lower end of the left tibia and the posterior border of the left calcaneal tuberosity (Fig. 3B). Bone ultrasound indicated synovitis and soft-tissue swelling in the lateral recess of the left ankle.

**Figure 4.**
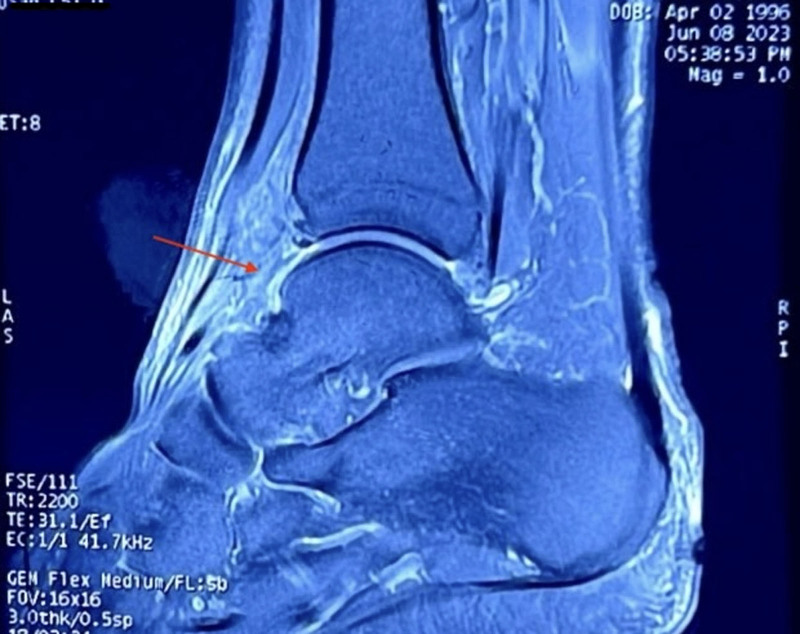
MRI sagittal imaging demonstrated ankle degeneration and mild thickening of the anterior talofibular ligament. MRI = magnetic resonance imaging.

Despite undergoing ice therapy, physiotherapy, and rehabilitation over the next year, the patient’s pain continued to escalate. In May 2023, further imaging at a local hospital showed slight bony hyperplasia at the lower end of the left tibia and the posterior edge of the left calcaneal tuberosity. Computed tomography (CT) scans confirmed cystic foci in the left calcaneus and talus, fluid in the joint cavity, and mild swelling of the surrounding soft tissues. MRI results suggested ankle degeneration and slight thickening of the anterior talofibular ligament (Fig. 4). A bone ultrasound revealed synovitis in the lateral recess of the left ankle, as well as swelling of the subcutaneous soft tissues. These findings led to the patient’s transfer to our hospital’s Department of Rehabilitation Medicine for additional diagnosis and treatment.

### 2.1. Intervention

Based on the evaluation of the patient’s clinical symptoms and assessment outcomes, a rehabilitation strategy is formulated, prioritizing areas such as pain, swelling, range of motion, strength, and proprioception. The following is an outline of the treatment modalities for each area.

During the first week, treatment focused on pain and swelling management. Muscle release techniques were applied to the gastrocnemius, soleus, tibialis anterior, peroneal longus, and tibialis posterior muscles, followed by 20-minute microwave therapy to alleviate pain and inflammation. Daily ultrasound therapy (20 minutes) and ankle pump exercises were utilized to control swelling. In the second week, interventions targeted range of motion improvement. Subtalar joint loosening techniques were applied, with continued microwave and ultrasound therapy as needed, along with ongoing ankle pump exercises. Weeks 3 and 4 emphasized strength and functional recovery. Isometric resistance exercises were introduced, focusing on the 4 directions of ankle movement (dorsiflexion, plantarflexion, inversion, and eversion), with 3 sets of 15 repetitions. Light functional activities, including controlled weight-bearing, were also initiated. Weeks 5 and 6 focused on proprioception and advanced strengthening. Proprioceptive exercises, such as ankle letter-writing and single-leg squats, were implemented, along with progressive resistance and balance training, including single-leg drills on unstable surfaces, to enhance strength and coordination (Table 1).

**Table 1:** Timeline of the patient’s rehabilitation.

Week	Focus areas	Treatment modalities
1	Pain and swelling management	Muscle release techniques (gastrocnemius, soleus, and tibialis), 20 minutes microwave therapy, daily ultrasound therapy, and ankle pump exercises
2	Range of motion improvement	Subtalar joint loosening techniques, continued microwave and ultrasound therapy, and ankle pump exercises
3–4	Strength and functional recovery	Isometric resistance exercises (3 sets of 15 reps for dorsiflexion, plantarflexion, inversion, and eversion), controlled weight-bearing exercises
5–6	Proprioception and advanced strengthening	Proprioceptive exercises (ankle letter-writing, single-leg squats), progressive resistance, and balance training on unstable surfaces

### 2.2. Outcome

**Figure 5.**
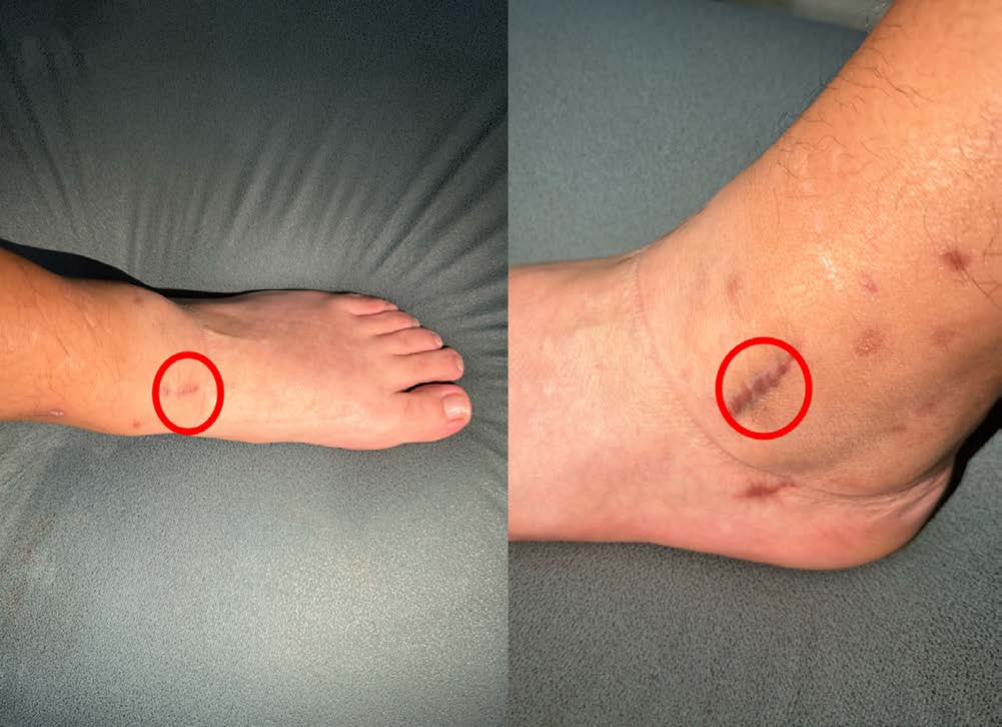
The points of the patient had pain in the joints and soreness in the muscles.

Following the initial treatment, the patient’s pain symptoms were temporarily alleviated, and the ankle’s range of motion was significantly improved. However, within 2 weeks of the patient’s discharge, the patient reported a recurrence of pain in the left ankle (Fig. 5), which progressively worsened, particularly at night, to the extent that standing became unfeasible. Due to the persistence and recurrence of symptoms, it was recommended that the patient undergo further diagnostic imaging, including MRI and CT scans.

**Figure 6.**
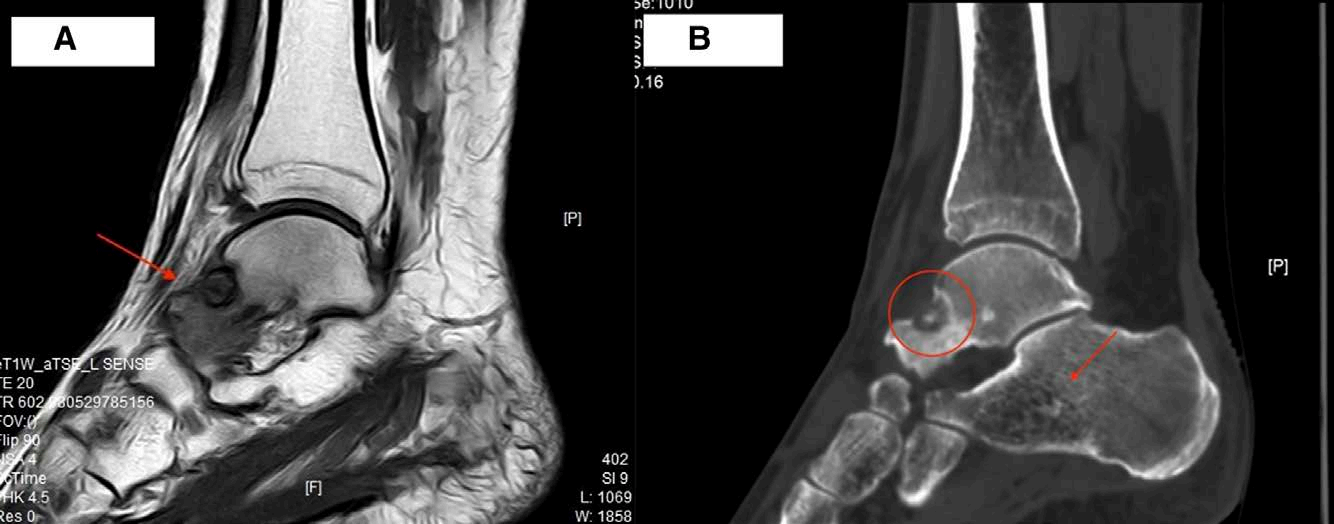
(A) MRI demonstrated localized bone marrow edema of the left talus and specific bone defects at the anterior superior border of the talus. (B) CT demonstrated an OO in the left talus, along with the formation of a calcaneal cyst and associated soft tissue swelling with effusion. CT = computed tomography, MRI = magnetic resonance imaging, OO = osteoid osteoma.

In August 2023, the patient underwent an imaging examination at our medical center. MRI revealed localized bone marrow edema in the left talus and a specific bone defect at the anterior upper edge of the talus. Additionally, a lipoma and was detected at the anterior border of the left calcaneus (Fig. 6A). CT revealed an OO in the left talus, along with the formation of a calcaneal cyst and associated soft-tissue swelling with effusion (Fig. 6B).

Subsequently, in September 2023, the patient underwent a complex surgical intervention under general anesthesia. This procedure included peripheral nerve transfer, joint debridement, resection of calcaneal and talus lesions, and tendon release. Postoperative management included the administration of anti-inflammatory and analgesic medications, as well as prophylactic measures against infection. The patient exhibited a stable postoperative course with no notable discomfort. The therapeutic interventions resulted in a gradual resolution of pain and swelling in the left ankle, culminating in a complete resolution. During the follow-up period of 2 months, the patient reported no significant discomfort associated with the left ankle joint. The patient underwent 3 functional assessments at different stages: before rehabilitation intervention, after the intervention, and during the 2-month follow-up period post-surgery. The assessments included evaluations of pain, swelling, ankle range of motion, and balance function (Table 2). Based on the evaluation results, the patient’s postoperative ankle function was significantly improved, and the pain and swelling were significantly reduced.

**Table 2:** Ankle function assessment.

Time	Pain	Swelling	Manual muscle testing		Ankle range of motion				Balance
	Visual Analogue Scale	Ankle circumference (cm)	Ankle dorsiflexor strength	Ankle plantar flexor strength	Dorsiflexion	Plantar flexion	Inversion	Eversion	Berg Scales
Preintervention assessment	4	32	4−	4	0°–10°	0°–42°	0°–8°	0°–4°	38
Postintervention assessment	2	29.5	4	4+	0°–17°	0°–53°	0°–16°	0°–6°	48
2-mo follow-up after surgery	0	28	5	5	0°–24°	0°–58°	0°–32°	0°–10°	56

## 3. Discussion

The clinical presentation of ankle OO is often misleading, leading to frequent misdiagnosis. This case emphasizes the challenges in diagnosing ankle OO, which can present with typical but nonspecific symptoms such as swelling, nocturnal pain, and limited range of motion. Early imaging, such as MRI, often reveals signs like edema and synovitis,^[9]^ which can easily be mistaken for more common conditions like ankle synovitis, particularly following a sports injury. In our case, initial treatments targeted synovitis, offering only temporary relief, and the absence of CT imaging delayed the correct diagnosis. MRI is known to sometimes obscure early tumor nests, complicating the diagnosis further. As shown in Hosalkar et al’s study, the diagnostic accuracy of MRI for OO is only 3%, while CT achieves 67%, highlighting the importance of using a combination of imaging modalities.^[10]^ However, despite its higher diagnostic rate, CT alone is not entirely reliable, emphasizing the need for a comprehensive diagnostic approach, especially in the early stages. In our case, the definitive diagnosis was achieved only after repeated failures of symptomatic treatments, underscoring the value of multidisciplinary consultations in such complex presentations.

This case illustrates the limitations of relying solely on imaging for OO diagnosis and highlights the critical role rehabilitation therapists play in multidisciplinary care. Collaboration between radiologists, orthopedic surgeons, oncologists, and rehabilitation professionals ensures that patients with persistent, unexplained symptoms receive timely and accurate diagnoses. Although ankle OO is rare, its distinct clinical feature nocturnal pain relieved by anti-inflammatory drugs should prompt further investigation, particularly when standard treatments fail. For rehabilitation therapists managing patients with persistent or worsening pain after ankle injuries, it is crucial to remain vigilant for the signs of talus OO. Instead of merely adjusting rehabilitation strategies, therapists should advocate for multidisciplinary evaluations to reassess the initial diagnosis. Accurate and early diagnosis of OO is essential to guide appropriate treatment, ensure patient recovery, and avoid unnecessary interventions.

## 4. Conclusion

Ankle OO is susceptible to misdiagnosis. Although CT and MRI are considered the gold standard for diagnosing OO, misdiagnoses remain relatively common in clinical practice. Rehabilitation therapists, who provide long-term treatment and evaluation, are uniquely positioned to monitor patient progress and understand their functional status. Consequently, they play a crucial role in diagnosing ankle osteoid tumors. Multidisciplinary teamwork, beyond just standardized imaging tests, offers a more timely and accurate diagnosis of such rare diseases. Rehabilitation therapists are essential in managing rare and easily misdiagnosed conditions by not only alleviating symptoms and improving function, but also ensuring accurate diagnosis and comprehensive treatment within a multidisciplinary team.

## Acknowledgments

We are particularly grateful to all the people who have given us help on our article.

## Author contributions

**Data curation:** Jincheng Xu.

**Supervision:** Shuming Yang.

**Writing – original draft:** Jincheng Xu.

**Writing – review & editing:** Shuming Yang, Yan Zhang.
